# Host-microbe interactions characterized by gene expression of cervical adhesion molecules, cytokines, and growth factors define the recurrence of bacterial vaginosis

**DOI:** 10.21203/rs.3.rs-7576831/v1

**Published:** 2025-09-23

**Authors:** Nichole Klatt, Erik Swanson, Courtney Broedlow, Emily Cherenack, Nicholas Nogueira, Christopher Basting, Pan Yue, Ashma Chakrawarti, Ty Schroeder, Ana Salazar, Lunarie Acosta, Patricia Raccamarich, Michael Gale, Lydia Fein, Maria Alcaide

**Affiliations:** University of Washington; Department of Surgery, University of Minnesota Medical School; University of Maryland; Duke University; University of Miami; University of Minnesota; University of Miami; University of Minnesota; University of Minnesota; University of Miami; University of Miami; University of Miami; University of Minnesota School of Medicine; University of Miami; University of Miami

## Abstract

Bacterial vaginosis (BV) is a common vaginal condition with a high recurrence rate after treatment. In this longitudinal multi-omics study, we integrated cervical microbial metatranscriptomics, host transcriptomics, cytokine profiles, and behavioral data to investigate factors driving BV recurrence in women from Miami-Dade county (N24). Recurrence at 6 months occurred in 46% of participants after metronidazole treatment. Recurrence was preceded by increased transcriptional activity of Gardnerella and Fannyhessea, enriched for glycogen and maltose metabolism and iron scavenging. Host transcriptomic analysis of cervical tissue revealed reduced CEACAM5–7 expression and increased IL6 and EREG, indicating impaired epithelial integrity and persistent inflammation. Cytokine–gene correlations and Bayesian mediation models identified CEACAM7 as a key mediator linking inflammation and microbial activity to recurrence. Intravaginal practices further amplified risk. These findings uncover for the first time dynamic host–microbiome disruptions that persist after treatment and reveals new targets for diagnostic and therapeutic strategies to reduce BV recurrence.

Bacterial vaginosis (BV) is the most common vaginal condition among women of reproductive age, affecting at least 30% of women globally^1–4^. BV is associated with significant clinical and public health consequences, including discomfort, obstetric complications, pelvic inflammatory disease, and increased risk of sexually transmitted infections including HIV^4–15^. Clinically, BV has traditionally been defined by Amsel’s criteria or Nugent scoring—clinical diagnostic methods that describe characteristic discharge, pH elevation, clue cells, and shifts in vaginal flora^16,17^. While useful for diagnosis in the clinical setting, these tools offer limited insight into the underlying microbiological and immunologic state of the cervicovaginal mucosa which may have more importance in disease. Increasingly, molecular profiling has revealed BV as a polymicrobial condition involving a shift from the optimal *Lactobacillus*-dominant microbiome to a diverse assemblage of non-optimal anaerobes, especially *Gardnerella spp*.^7,18–29^. However, current diagnostic frameworks still largely rely on binary classification (BV vs. normal), which fails to capture the condition’s heterogeneity or inform prognosis and treatment^30^.

A persistent challenge in BV management is recurrence. Although standard antibiotic treatment (typically metronidazole or clindamycin) is effective in resolving symptoms short-term, recurrence rates remain unacceptably high: up to 50% within 6 months and even higher by one year^20,26,31,32^. This high relapse rate suggests that existing treatments do not fully resolve either the host or the microbial factors sustaining non-optimal microbiome states. Currently, there are no validated biomarkers or stratification tools to identify which women are likely to experience recurrence, and little is known mechanistically about why some patients relapse repeatedly while others remain symptom-free after identical treatment with antibiotics.

Several non-mutually exclusive mechanisms have been proposed to explain recurrence—persistent microbial biofilms, failure to re-establish a Lactobacillus-dominant microbiota, host immune dysregulation, and reinfection from sexual partners^1–3,26,31,32,32–34^. Yet none of these theories fully account for the variability in treatment outcomes, and clinical trials targeting any single factor have yielded limited success^34–37^. The complexity of BV recurrence likely reflects a nuanced interplay of microbial, host, and behavioral influences, but the BV research field still lacks an integrated framework for studying these interactions over time to decipher BV recurrence trajectory.

To address this knowledge gap, we conducted a longitudinal, multi-omics investigation of BV recurrence following antibiotic treatment. By integrating the cervical transcriptome, cytokine profiles, cervical microbial transcriptome, and biobehavioral metadata, this study identified biomarkers predicting BV recurrence, and revealed connections between host inflammatory gene networks and microbial factors that drive BV recurrence.

## RESULTS

Cytokine concentration correlations with microbiome

Bayesian modeling – microbiome synthesis

### Framing the results

**Figure 1:**
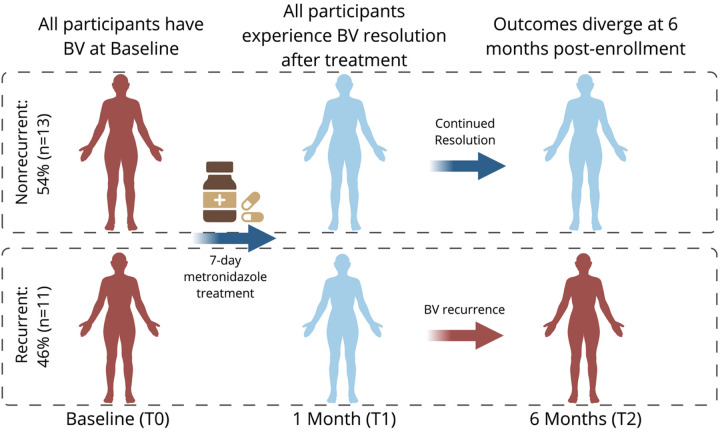
Comparison of participant’s recurrent vs non-recurrent BV trajectories. A longitudinal schematic of cohort groupings and treatment response. Red indicates BV diagnosis by Amsel’s criteria, blue indicates BV resolution. Created with Biorender.

Our study compared recurrent and noncurrent BV. All participants had BV by Amsel’s criteria at baseline and BV resolution 1-month after metronidazole antibiotic treatment. BV recurrence status was determined 6-months post-treatment, when 46% of participants were diagnosed with BV by Amsel’s criteria again (recurrent) and 54% remained BV negative (nonrecurrent). This design allowed us to assess the difference between the recurrent and nonrecurrent BV trajectory at each stage of progression rather than as a single comparison between BV and control participants (Fig. 1).

### Cervical microbiome diversity and differential gene expression reveal marked differences between BV recurrence groups

**Figure 2:**
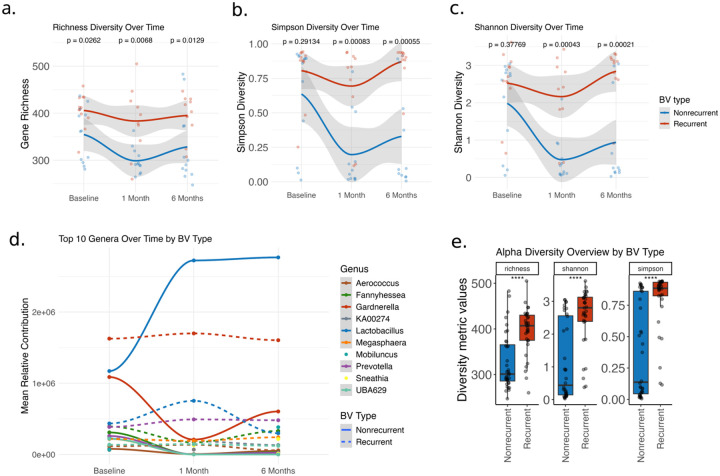
Alpha diversity dynamics and taxonomic contribution over time by BV recurrence status. (a–c) Longitudinal changes in microbial alpha diversity metrics—(a) gene richness, (b) Simpson diversity, and (c) Shannon diversity—calculated from the cervical bacterial metatranscriptome at three timepoints: baseline, 1-month post-treatment, and 6-months post treatment. Participants are stratified by BV recurrence status at 6-months (nonrecurrent: blue; recurrent: red), with all participants Amsel-positive at baseline and Amsel-negative at 1 month. Shaded areas represent 95% confidence intervals; p-values reflect Wilcoxen comparisons between recurrence groups at each timepoint. (d) Mean relative contribution of the top 10 most abundant bacterial genera over time, stratified by recurrence status. Solid lines represent nonrecurrent BV; dashed lines represent recurrent BV, and colors represent genera. (e) Overview of alpha diversity across all timepoints, comparing recurrent and nonrecurrent groups. Boxplots show distributions of richness, Shannon, and Simpson indices; **** indicates p < 0.0001 by Wilcoxen test.

Our functional genomics sequencing data demonstrate that cervical microbiome alpha diversity as measured by gene richness, Shannon gene diversity, and Simpson gene diversity significantly differs between women with recurrent and nonrecurrent BV (Fig. 2a–c). Gene richness was significantly different between BV recurrence groups at baseline (p < 0.05), and that difference increased after treatment at 1 and 6-month follow-up samples (p < 0.02). Shannon and Simpson metrics showed the same pattern, with an even wider divergence in alpha diversity after treatment at 1 and 6-month follow-up visits (p < 0.001, Fig. 2b–c).

Collapsing expression data to the taxa level demonstrated dynamic longitudinal differences in the transcriptional activity of key BV-associated microbes. Gene expression between BV groups for *Lactobacillus*, *Gardnerella, Fannyhessea, Sneathia, Prevotella*, and *Megasphaera* was significantly different by 1-month post-treatment (p = 0.03–0.0003, Fig. 2d and supplemental Fig. 2b)

**Figure 3:**
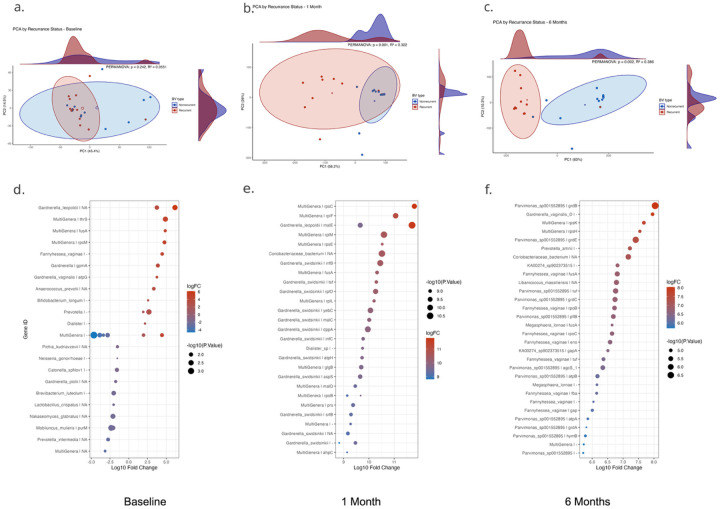
Bacterial community structure and differentially expressed bacterial genes over time by BV recurrence status. (a–c) Principal component analysis (PCA) of bacterial metatranscriptomic profiles at (a) baseline, (b) 1-month post-treatment, and (c) 6-months post-treatment, stratified by recurrence status (nonrecurrent: blue; recurrent: red). PERMANOVA R^2^ and p-value is listed on each plot for BV-group comparison. Shaded ellipses represent 95% confidence intervals for each group; density plots show sample distributions along each PC axis; open circles show group centroids. PCA axis loadings are available in supplementary data Figure 3. (d–f) Dot plots of differentially expressed bacterial genes (recurrent vs nonrecurrent) at (d) baseline, (e) 1 month, and (f) 6 months. Dot size represents – log_10_(adjusted p-value), and color reflects log_10_ fold change (logFC) scaled per-timepoint. Gene names include genus/species annotations where available; “MultiGenera” indicates genes indistinguishably mapping to multiple taxa. Numerous Gardnerella- and Fannyhessea-associated genes are enriched in recurrent participants, particularly at later timepoints, notably in the glycogen and maltose pathways.

Shifting to community composition, the differences we observed in alpha diversity were paralleled by beta diversity differences in the bacterial transcriptome as measured by Principal Components Analysis (PCA) and Permutational Multivariate Analysis of Variance (PERMANOVA) (Figs. 3a–c). Beta diversity in the cervical metatranscriptome at baseline was not significantly different between BV recurrence groups, but was significantly different after treatment with a PERMANOVA R^2^ above 0.3 and p-values ≤ 0.002 for both 1 and 6-month follow-up visits (Fig. 3b–c). PCA loadings from the first two axes show that *Gardnerella* and *Fannyhessea* gene expression, notably glycogen (*glg*) and maltose (*mal*) genes from *Gardnerella* heavily contribute to the beta diversity differences we observed, especially at 1-month post-treatment (supplemental Fig. 3).

Probing the microbiome differences between BV types with Limma-Voom differential expression analysis defined a cervical microbiome with altered mucosal carbon utilization, iron scavenging, biofilm formation, hypoxia resistance, and off target antimicrobial resistance (Fig. 3d–f). Specifically, glycogen and maltose metabolism, and gentamycin resistance operons (*glg, mal, grd*) each had significantly higher expression in recurrent BV. Expression of individual genes also stood out, with the iron-regulated cell surface anchor sortase B (*srtB*), iron chelating Elongation Factor G (*fusA*), and Alkyl Hydroperoxide Reductase (*ahpC*) all increased in the recurrent BV microbiome. Together, these data reveal a distinct functional divergence in the microbiome of women with recurrent BV after antibiotic treatment.

### The cervical transcriptome of tissue remodeling genes differentiates recurrent and nonrecurrent BV

**Figure 4:**
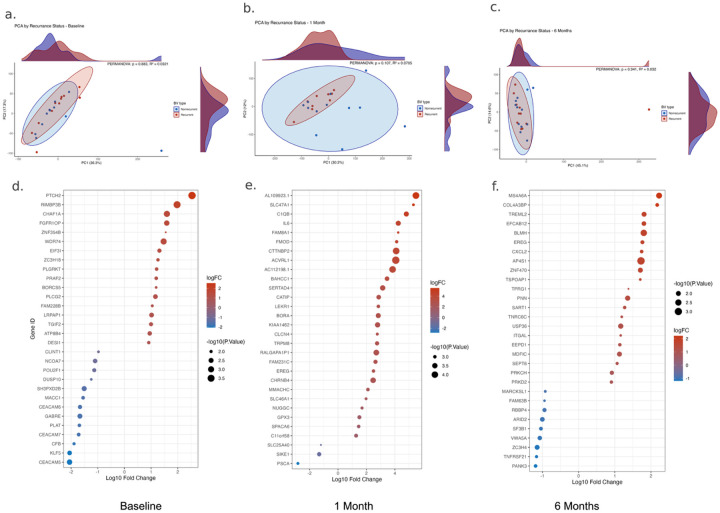
Cervical transcriptome differences by BV recurrence status over time. (a–c) Principal component analysis (PCA) of host transcriptomic profiles (human gene expression) at (a) baseline, (b) 1-month post-treatment, and (c) 6-months post-treatment, comparing recurrent (red) and nonrecurrent (blue) participants. PERMANOVA R^2^ and p-value is listed on each plot for BV-group comparison. Ellipses represent 95% confidence intervals for each group; marginal density plots show sample distributions along each principal component. (d–f) Dot plots showing differentially expressed host genes between recurrence groups at (d) baseline, (e) 1 month, and (f) 6 months. Dot size reflects −log_10_(adjusted p-value), and color indicates log_10_ fold change (logFC) scaled per-timepoint. Notably, immunoregulatory and epithelial integrity genes appear differentially expressed across timepoints.

In the paired cervical transcriptome from each participant, PCA and PERMANOVA did not show significant overall differences in gene expression between BV types (Fig. 4a–c). However, differential gene expression analysis with Limma-Voom showed that individual genes were significantly distinct (Fig. 4d–f). Differentially expressed cervical genes between BV recurrence groups frequently implicated tissue remolding, inflammation, and wound healing pathways. Our most notable differential gene expression findings were significantly decreased expression of carcinoembryonic antigen-related cell adhesion molecules (CEACAM5, CEACAM6, and CEACAM7) at baseline, increased interleukin-6 (IL-6), epiregulin (EREG), and activin receptor-like kinase 1 (ACVRL1) expression at 1-month, and a continued increase in EREG expression at 6-months (Fig. 4d–f). This gene expression pattern implicates barrier integrity attenuation as a biologically plausible mechanism for BV recurrence, and indicates that this network of genes could be considered as potential targets for therapeutic interventions against BV..

### Cervical and bacterial functional pathways differentiate BV recurrence groups

**Figure 5:**
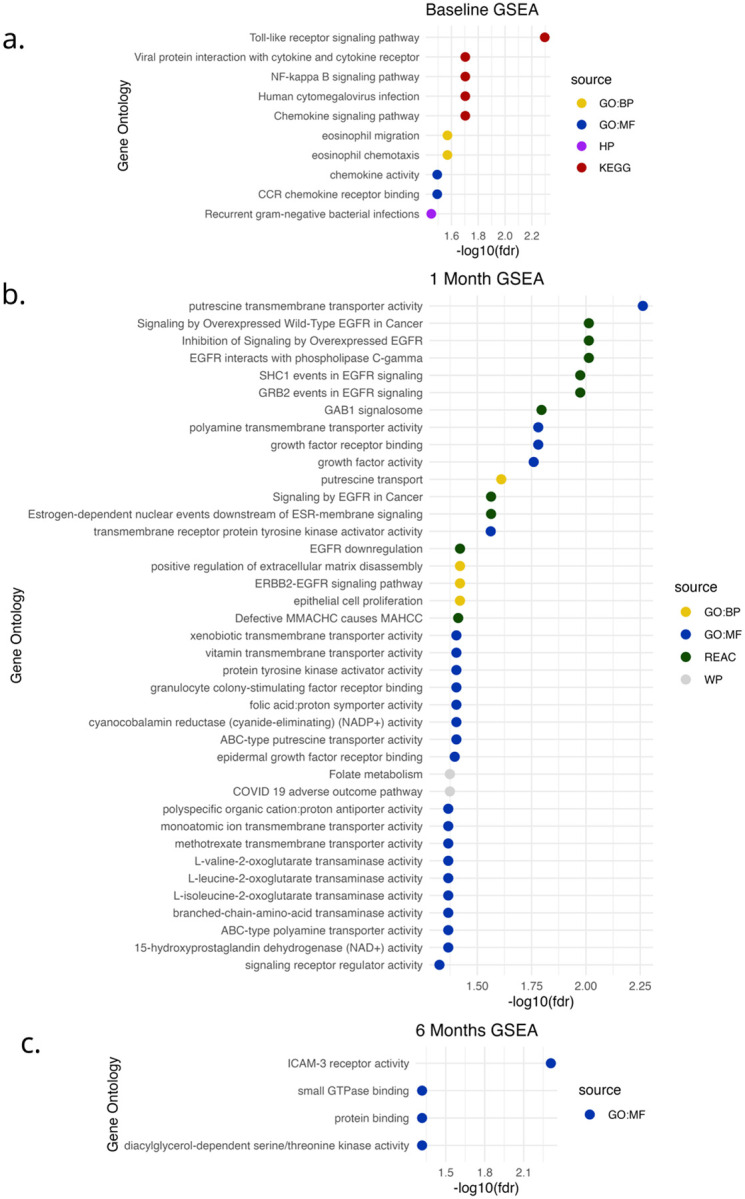
Longitudinal gene set enrichment analysis (GSEA) of host cervical transcriptomes stratified by BV recurrence status. Dot plots show significantly enriched host gene ontology (GO), pathway, and molecular function terms at (a) baseline, (b) 1-month post-treatment, and (c) 6-months post-treatment, based on differential expression between recurrent and nonrecurrent participants based on Amsel BV criteria. Enrichment was performed using gene ontology biological processes (GO:BP), molecular functions (GO:MF), KEGG pathways (KEGG), Reactome pathways (REAC), Human Phenotype (HP), and WikiPathways (WP), with results filtered at FDR < 0.05. Color indicates source ontology, and dot position reflects significance (–log_10_[FDR]). Key findings include baseline enrichment of immune-related pathways (e.g., NF-κB signaling, chemokine signaling) in participants who would later experience recurrence, strong EGFR signaling and transporter-related activity at 1-month, and limited but persistent signaling differences at 6 months.

**Figure 6:**
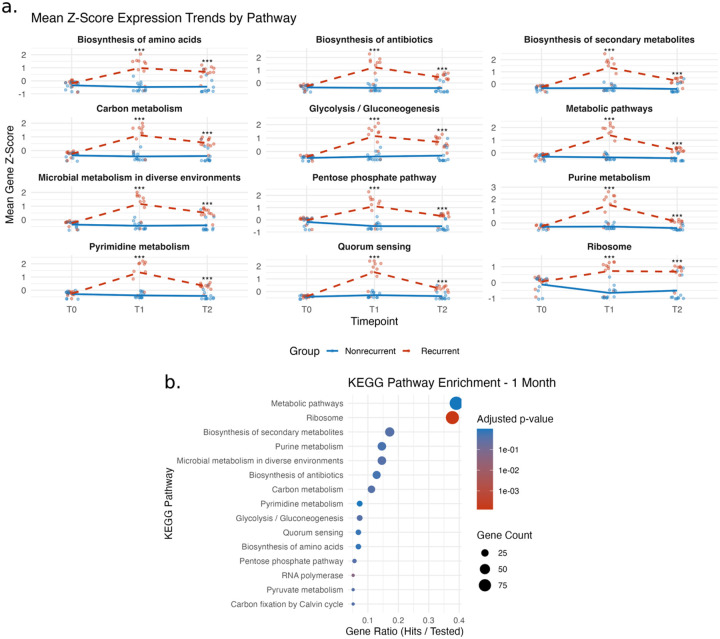
Longitudinal KEGG pathway expression and enrichment in cervical bacterial metatranscriptome by BV recurrence status. (a) Line plots showing mean Z-score trends for selected KEGG pathways significantly enriched in bacterial transcripts, plotted over three timepoints: baseline (T0), 1 month (T1), and 6 months (T2). Participants were stratified by BV recurrence status at 6 months (nonrecurrent: blue solid line; recurrent: red dashed line). Pathways were selected based on relevance to metabolism, microbial adaptation, and quorum sensing. Each point represents the mean normalized expression (Z-score) of all genes in the pathway per participant; asterisks indicate significance levels comparing recurrence groups at each timepoint (*p < 0.05; **p < 0.01; ***p < 0.001, adjusted). (b) Dot plot of KEGG pathway enrichment based on differentially expressed bacterial genes at 1 month (T1) between recurrence groups. The x-axis represents the gene ratio (hits/total tested), dot size reflects the number of genes contributing to the enrichment, and color indicates adjusted p-value. Key enriched pathways include ribosome biogenesis, diverse metabolic processes, and microbial signaling systems such as quorum sensing.

Interrogating gene expression at the functional level using Kyoto Encyclopedia of Genes and Genomes (KEGG) and gene ontology (GO) enrichment analysis (using *enricher* and *gost* in R, respectively) revealed significant pathway enrichments and the same high-level pattern we observed in alpha and beta diversity results – namely that significant differences exist in host and microbe gene expression between BV recurrence groups, even when participants have the same Amsel’s BV status (Figs. 5 and 6).

Interestingly, women with recurrent BV had elevated expression of genes in the *viral infection response*, *chemokine signaling*, *eosinophil chemotaxis* and *eosinophil migration*, and *recurrent gram-negative infection* pathways at baseline – all pointing towards higher immune activation relative to women with nonrecurrent BV (Fig. 5a). Notably, we also observed significantly more cervical human papilloma virus (HPV) and herpes virus transcripts in recurrent BV, but at 1-month post treatment rather than at baseline (supplemental Fig. 4). We also observed significantly increased Trichomonas expression at women with recurrent BV at the 6-month follow-up time point, extending the connection between BV recurrence susceptibility and sexually transmitted infections (supplemental Fig. 5).

Our GO pathway analysis at of cervical tissue gene expression 1-month post-treatment revealed a clear gene expression pattern implicating the EREG (a key upregulated gene) receptor pathway *epithelial growth factor receptor* (EGFR). We identified seven different EGFR pathways enriched in women with recurrent BV. Also interesting were two putrescine transport pathways upregulated in recurrent BV (Fig. 5b). Surprisingly, we saw few pathway enrichments between BV groups at 6-months post treatment (Fig. 5c).

Cervical transcriptome pathway enrichments were accompanied by bacterial KEGG pathway enrichments in the cervical metatranscriptome (Fig. 6). Unsurprisingly, enriched bacterial KEGG pathways included *metabolic pathways* and *glycolysis/gluconeogenesis*, both of which include genes from the maltose and glycogen operons (Fig. 6). However, the continued pattern BV groups sharply diverging at 1-month post-treatment was more prominent that the specific bacterial pathway enrichments (Fig. 6a). Our cervical transcriptome data reveal functional differences in women with recurrent BV that center on tissue damage response and barrier maintainence.

### Cytokine protein levels in cervicovaginal lavage samples correlate with differential gene expression

**Figure 7:**
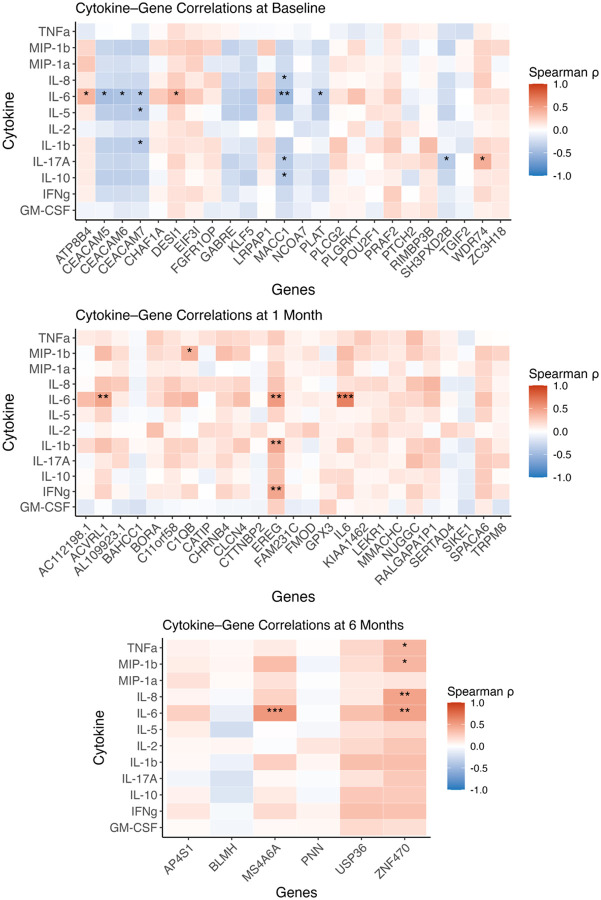
Spearman correlations between cervical cytokine protein concentration and host gene expressionacross the study period. Heatmaps show pairwise Spearman ρ values between 13 cytokines (rows) and selected human genes (columns) at three timepoints: baseline (top), 1-month (middle), and 6-months (bottom) post-treatment. Color scale reflects direction and strength of correlation (red = positive; blue = negative). Asterisks indicate adjusted p-values: *p* < 0.05 (*), p < 0.01 (**), p < 0.001 (***). At baseline, significant negative correlations are observed between CEACAM5/6/7 and several inflammatory cytokines (e.g., IL-6, IL-1β). At 1-month, strong positive associations emerge between IL-6, IL-1b, IFN-γ and *EREG*. By 6 months, IL-6 shifts to strongly correlated with *PNN*, *USP36*, and *ZNF470*. These results highlight dynamic host immune–epithelial gene relationships across BV resolution and recurrence.

Given the clear pattern of differences between BV groups we integrated our functional genomics datasets by using Spearman correlations to associate cytokine protein levels with differentially expressed human and bacterial genes. This analysis yielded many significant and revealing connections (Fig. 7, supplemental Figs. 7–13). We found significant correlations between IL-6 protein levels and several differentially regulated genes including CEACAM5, CEACAM6, CEACAM7, EREG, ACVRL1 and MACC1. CEACAM7 and EREG particularly stood out by correlating with several cytokines – CEACAM7 negatively with IL-6, IL-5, and IL-1β and EREG positively with IL-6, IL-8, IL-17α, and IL-10 (Fig. 7a–b). Simple comparisons of cytokine levels between recurrence groups yielded no additional insight (Supplemental Figs. 14–16).

Dozens of bacterial genes were significantly correlated with cytokine protein levels, especially IL1β, TNFα, and GM-CSF at 1-month and 6-month time points (supplemental Fig. 11–13). We clustered the gene-cytokine associations with Clusters of Orthologous Genes (COG) groupings, showing that bacterial genes assigned the COG category *translation, ribosomal structure, and biogenesis* were most commonly associated with cytokine levels. That finding indicates that bacterial metabolic activity linked with bacterial colonization and growth directly links with inflammation.

Moreover, using spearman analysis of cervical gene expression and microbial gene revealed that at 6-months post-treatment Ubiquitin-Specific Peptidase 36 (USP36) was positively associated with anaerobic bacteria, and Pinin (PNN) was negatively associated with *Lactobacillus iners* (supplemental Fig. 16), while no significant correlations occurred at baseline or 1-month post-treatment. In all, we found that the individual biomarkers of recurrent BV we identified are correlated with cervicovaginal inflammation, providing additional targets for intervention to cure BV.

### Integrating multiomics with Bayesian modeling shows conditional and mediation effects between host and microbiome factors

**Figure 8:**
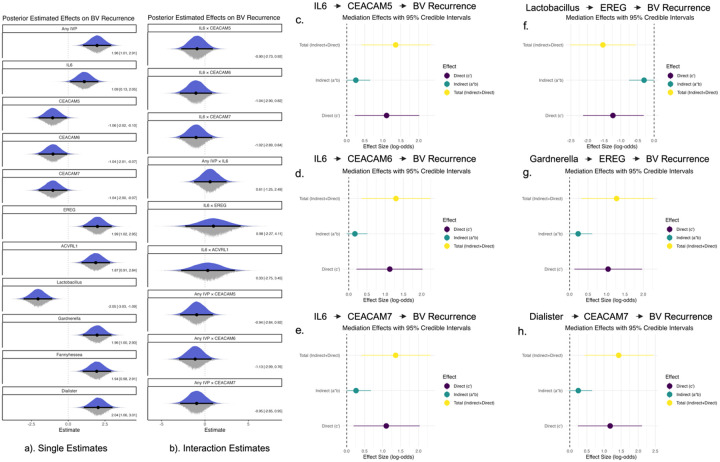
Bayesian posterior estimates and mediation models of BV recurrence. Panel (a) shows posterior distributions for single-variable models estimating associations with BV recurrence, with median and 95% credible intervals. Variables include behavioral (Any IVP), host (IL6, EREG, ACVRL1, CEACAM5–7), and microbial (Lactobacillus, Gardnerella, Fannyhessea, Dialister) features. Panel (b) shows posterior estimates for pairwise interaction terms tested between select host, microbial, and behavioral variables. All models were fit with weakly informative priors and include controlled for multiple sampling. Panels (c–h) show posterior estimates with 95% credible intervals for mediation models assessing indirect (a*b), direct (c’), and total effects of host and microbial predictors on BV recurrence. (c–e) represent models testing mediation by CEACAM5, CEACAM6, and CEACAM7 on the effect of IL6 on BV recurrence. (f–g) show EREG as a mediator of *Lactobacillus* and *Gardnerella*. (h) shows the mediation of CEACAM7 on the effect of *Dialister* and BV recurrence. Effect sizes are in log-odds. Full posterior parameters for these and additional mediations are found in supplemental Figures 17–22.

**Figure 9:**
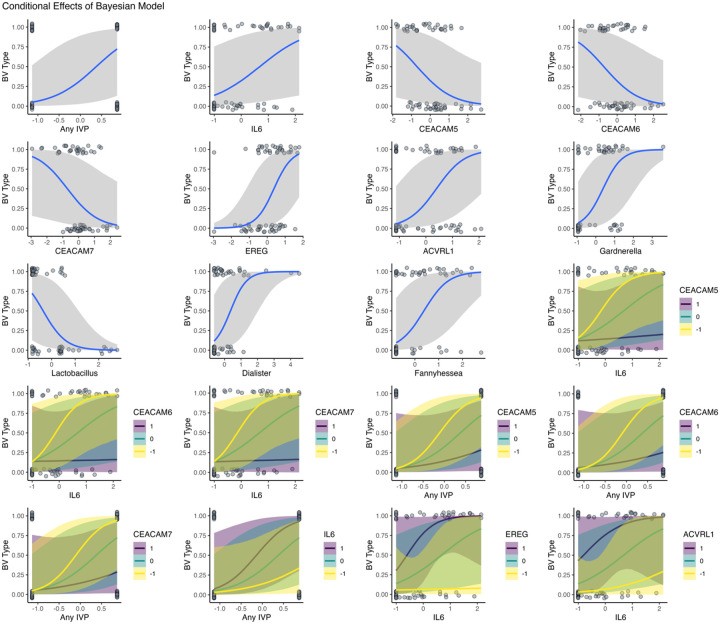
Conditional effects of host and microbial features on BV recurrence based on Bayesian logistic regression. Plots show the estimated probability of BV recurrence (y-axis) as a function of each covariate (x-axis), holding all other variables at their means. Top three rows primarily show marginal (main) effects of individual predictors, including intravaginal practices (IVP), genes (IL6, EREG, ACVRL1), CEACAM family members, and key microbial taxa (*Lactobacillus*, *Gardnerella*, *Fannyhessea*, *Dialister*). Blue lines indicate posterior mean predictions; shaded areas represent 95% credible intervals. Observed values (jittered) are overlaid as points. The bottom three rows show conditional interaction effects, where the impact of IL6 or IVP is modulated by host gene expression. Line color represents centered and scaled levels of the moderator variable (purple: low, green: average, yellow: high). Notable interactions include stronger recurrence risk with high IL6 and low CEACAM expression, and additive risk with both IVP presence and low CEACAM levels. These conditional plots illustrate the non-linear and context-dependent effects of host immune markers and microbiome features on BV recurrence probability.

We next employed Bayesian methods to build integrative models connecting each individual facet of this study – host gene expression, bacterial gene expression, behavior, and cytokine quantification. We found statistically credible positive effects of IL6, EREG, ACVRL1, intravaginal practices (IVPs), *Gardnerella*, *Fannyhessea*, and *Dialister* on BV recurrence, and statistically credible negative effects of each CEACAM gene and *Lactobacillus* on BV recurrence (Fig. 8a, credible ≈ significant – see methods for explanation of Bayesian terminology). Our interaction models reveal directional trends of pairwise parameter interactions, but with only moderate confidence (Fig. 8b). Extending the analysis to model the conditional effects of selected parameters, we found that low CEACAM gene expression is only associated with recurrence if IL6 is moderate or highly expressed. Furthermore, we found that high IL6 expression is only associated with recurrence at moderate or high EREG expression levels (Fig. 9). We also show that IVPs are associated with BV recurrence conditionally with lower CEACAM expression and higher IL6 concentration (Fig. 9). These data reveal that individual associations of biomarkers with BV recurrence are conditional on the expression level of other biomarkers – namely the IL6-CEACAM-EREG axis.

**Figure 10:**
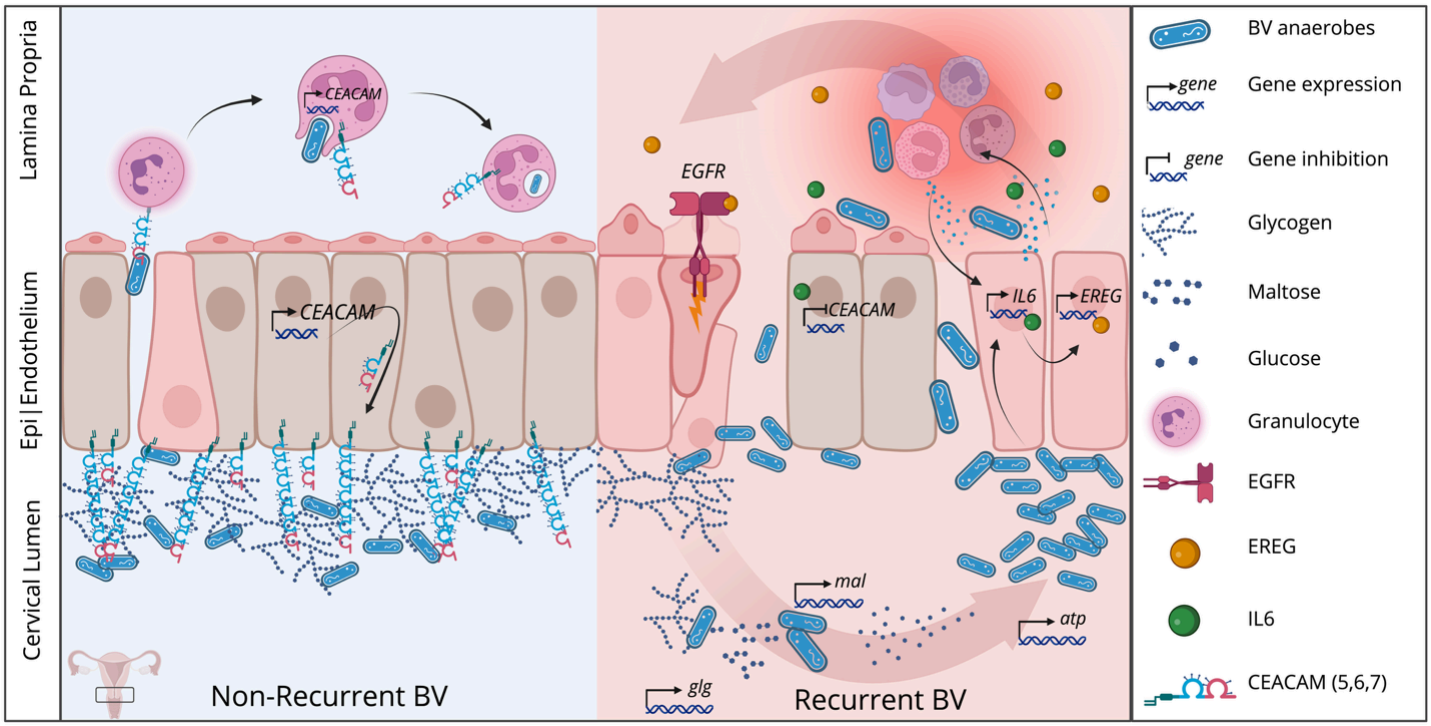
Proposed model of recurrent BV cervical dynamics. These findings require confirmation by future *in vitro* studies, but the proposed model cervical interactions is consistent with and informed by our data and modeling. The right panel illustrates how treatment-responsive BV resolution is aided by increased CEACAM expression and decreased IL6 and EREG/EGFR expression. The left panel shows how BV anaerobes (notably *Gardnerella* and *Dialister* species) interact with modified host gene expression in a reciprocal fashion – being both fed by increased tissue damage and glycogen release and feeding the inflammation feedback loop. glg = glycogen operon expression, mal = maltose operon expression, atp = atpase operon expression. Created with Biorender.

Finally, we extended our multiomics integration by explicitly testing directional mediation paths between a distilled set of factors including the most promising cytokine level (IL6), gene expression (CEACAMs, EREG), bacterial activity (*Lactobacillus, Gardnerella, Fannyhessea, Dialister*) and BV recurrence. Our mediation analysis revealed modest but statistically credible mediation relationships for the causal paths IL-6-CEACAM-BV (for CEACAM 5 and 7), *Dialister*-CEACAM7-BV, *Gardnerella*-EREG-BV, and *Lactobacillus*-EREG-BV (Fig. 7c–h, supplemental Figs. 17–22). Our results suggest a combination of host behavior (IVPs) and underlying cervical gene expression (CEACAMs, EREG) primes the cervical mucosa for microbial disturbance and perpetuates a cycle of elevated inflammation (IL-6), tissue damage, and increased anaerobic microbial activity (*Gardnerella, Fannyhessea*). These results provide a new level of granularity to our understanding of cervical microbiome dynamics in recurrent BV, as well as completely novel mechanistic insights linking human gene expression to recurrent BV, and provides targets for therapeutic intervention to cure BV and prevent recurrence (Fig. 10).

## DISCUSSION

This longitudinal study provides a novel, integrated perspective on BV recurrence, which has been poorly understood. By simultaneously profiling the cervical microbial metatranscriptome, host transcriptome, local cytokines, and behavioral factors, we uncovered coordinated host–microbe dysregulations distinguishing women with recurrent BV from those who maintain symptom resolution post-treatment. This comprehensive approach extends existing perspectives by illustrating how specific microbial activities associate with host responses in the critical post-treatment window.

Women with recurrent BV exhibited markedly different microbial trajectories following treatment, consistent with, but expanding on past research that assessed treatment response^20,26^. Despite all participants on our analysis being Amsel’s-negative at the 1-month visit, women with recurrent BV showed more diverse microbial transcription characterized by early reactivation of *Gardnerella* and *Fannyhessea* activity, contrasting sharply with non-recurrence cases. *Gardnerella* and *Fannyhessea* are archetypal taxa previously linked to BV recurrence^19,21,22,38^, but our study uniquely identifies metabolic activity months before clinical relapse and isolates the characteristics of recurrent BV from incidental BV. Our metatranscriptomic analysis revealed enrichment in glycogen and maltose metabolism, iron scavenging, biofilm formation, and antibiotic resistance pathways in recurrence-prone microbiomes. Both human and microbial amylases are known to liberate maltose from epithelial glycogen^39–42^, linking epithelial disruption and cell sloughing with microbiome metabolism activation and BV recurrence. Our data confirmed elevated expression of glycogen metabolism pathways (*mal* and *glg*) in conjunction with iron chelating (fusA)^43^ and iron-dependent cell wall modifications associated with biofilm formation in *Clostridium* (srtB)^44,45^ in *Gardnerella* and *Fannyhessea*. These mechanisms are widely implicated in pathogen survival^46,47^, but to our knowledge this is the first report of their association specifically with BV, supporting a model in which iron-dependent biofilm development is a key facet of recurrence. That idea has surfaced before in BV literature, but with mixed effects^48–50^. Our data adds weight to the most promising of those studies which found a modest protective effect of iron-binding lactoferrin in preventing recurrent BV. This suggests a model where incomplete restoration of *Lactobacillus* dominance, driven by iron scavenging, glycogen and maltose-fed anaerobes, contributes to BV recurrence. These data provide clear mechanistic targets for validation and future therapeutic intervention to prevent BV recurrence.

### Host Mucosal Immune Response in Recurrence

Parallel profiling of host gene expression revealed distinct cervical trans signatures in recurrence-prone women, notably reduced CEACAM5–7 expression coupled with increased inflammatory expression (IL6), EREG transcripts, and EGFR pathway activation. The CEACAM family is a group of epithelial adhesion molecules implicated in both barrier integrity and immune signaling^51–54^. Interestingly, past research shows that CEACAM3 and 6 are involved in innate pathogen detection by granulocytes, providing a clear mechanism in BV beyond epithelial dynamics^53^. We show here for the first time that three different CEACAM genes (5,6, and 7) are all downregulated in recurrent BV, potentially reflecting compromised epithelial-mesenchymal transition, mucosal barrier function, and phagocytosis^55–57^. The elevated IL6 and epiregulin expression we observed also suggest persistent mucosal inflammation and active epithelial remodeling after antibiotic treatment^58,59^. This imbalanced immune equilibrium, characterized by lower CEACAM expression, heightened inflammation, and tissue remodeling likely creates conditions favoring BV pathogen persistence and re-emergence in several ways, both directly by reducing pathogen detection, and indirectly by freeing microbial resources via dysregulated epithelial damage response.

### Host–Microbiome Correlations and Mechanistic Links

Besides identifying novel human and microbial genes associated with BV, we leveraged the strengths of both Bayesian and frequentist methods to integrate those individual findings in statistically testable causal models. Our integrated analysis revealed strong positive correlations between IL6 and epiregulin, both of which are associated with mucosal inflammation and repair. CEACAM5–7 negatively correlated with inflammation markers including IL6, suggesting a protective role in maintaining mucosal homeostasis. Furthermore, Bayesian mediation modeling indicated that CEACAM7 specifically plays a critical mediating role *between* microbial and immune factors and BV outcomes, pivotally influencing BV recurrence outcomes. Likewise, our conditional effects modeling shows that the impact of IL6 on BV recurrence is minimal if CEACAM expression is high, whereas at medium to low CEACAM expression levels, IL6 is a strong predictor of recurrence. These data suggest that IL6 and *Gardnerella* relate to BV in complex, context dependence ways, and that CEACAM genes are key to these associations

### Predictive Markers of Recurrence

Our multi-omic approach identified candidate predictive biomarkers for BV recurrence, notably microbial taxa (*Gardnerella*, *Dialister*), host immune factors (IL6, epiregulin), and behavioral variables (IVPs). Elevated IL6 and epiregulin post-treatment were sensitive markers for recurrence risk, highlighting ongoing mucosal inflammation as a critical determinant. Extending past findings^1^, we demonstrated that behavioral factors interact with immune factors (CEACAMs) to amplify recurrence risk. IVPs were associated with BV recurrence, but that association was conditional on CEACAM expression. At high CEACAM expression levels, IVPs were much less associated with recurrence. This combined biomarker approach could enhance predictive ability and suggests targets for personalized monitoring and prevention of BV recurrence.

Context with STIs and Viral Co-factors

Interestingly, we also demonstrated that recurrent BV cases were associated with expression of viral and protozoan STI co-infections including HPV, HSV, and *Trichomonas vaginalis*, possibly due to bidirectional interactions exacerbating mucosal inflammation and microbial dysbiosis. Considerable past research has addressed on the association of BV with STIs, but usually focus on BV as a risk for STIs, rather than the reverse^11,12,15,27,60^. The temporal nature of our data suggest that relationship could be more bidirectional than previously thought. We only found increased transcription of HPV and herpes virus during in recurrent BV during symptom remission, implicating those STIs as recurrence triggers, and that antibiotic use may allow for an environment more susceptible to STIs.

### Summary, limitations, and directions

Our study’s moderate sample size somewhat limits broad generalizability, and causality cannot be definitively established given our observational design. Although integrated analyses and Bayesian modeling provided mechanistic insights and causal inference, experimental validation of the identified pathways is essential. Limitations notwithstanding, our findings highlight multiple potential intervention points: reducing mucosal inflammation via IL-6 antagonists, enhancing epithelial barrier integrity by increasing CEACAM expression, modifying detrimental IVPs, and targeting microbial biofilm and metabolic pathways using maltose transport inhibitors or decoy alcohols. Combined with new probiotic interventions recently made available^34,35^, these strategies may help restore *Lactobacillus*-dominated microbiomes and merit further exploration. Our integrative multi-omic approach provides a foundation for precision-medicine strategies aimed at achieving durable BV cures and improving women’s reproductive and sexual health outcomes worldwide.

## METHODS

### Study design and participant recruitment

We analyzed samples from the Women, HIV, Immunology, Microbiome and Sexual Health (WHIMS) longitudinal cohort, which investigates biological and behavioral determinants of recurrent bacterial vaginosis (BV) and HIV risk. HIV-negative, cisgender women aged 18–45 residing in Miami-Dade County, Florida were enrolled between January 2019 and January 2024. Eligibility required sexual activity within the past three months and excluded pregnancy, recent antibiotic or STI treatment (within two months), immunosuppression, or prior cervical surgery. Participants were recruited via registries, clinics, and community outreach. All procedures were conducted at the University of Miami Infectious Diseases and CFAR clinical research unit. Informed consent was obtained from all participants under protocols approved by the University of Miami IRB (protocol #20180758), in accordance with the Declaration of Helsinki.

### Sample collection and processing

Participants completed baseline, 1-month, and 6-month follow-up visits that included biobehavioral surveys, gynecological exams, and BV testing using Amsel’s criteria. Cervical cytobrush samples were collected at each visit and stored in RNAlater (Thermo Fisher Scientific), at − 80°C. BV was diagnosed when at least three of four Amsel criteria were present (discharge, pH > 4.5, clue cells, positive KOH whiff test).

From the WHIMS cohort (n = 121), a subset of 24 participants (70 samples) was selected based on BV diagnosis at baseline, resolution at 1 month, and availability of high-quality cervical transcriptome, microbial metatranscriptome, and cervicovaginal lavage cytokine data. RNA was extracted using the RNeasy HT kit (Qiagen). Ribosomal RNA was depleted (RiboZero Plus, Illumina), and libraries were prepared and sequenced on an Illumina NovaSeq S4 platform (2 × 150 bp) at the University of Minnesota Genomics Center. Trimmomatic^61^ was used to remove adaptors and low-quality reads (Phred score < 30). Samples with < 3 million post-filter reads were excluded.

Reads were separated into human and microbial components using Bowtie2^62^. Human reads were mapped to the GRCh38^63^ human reference genome using kallisto^64^ and imported into R with tximport to compute length-scaled transcripts per million (TPM). Microbial reads were mapped to the VIRGO2^65^ vaginal metagenome catalog and similarly imported into R for TPM quantification.

### Microbial community diversity and taxonomic profiling

Alpha diversity (richness, Shannon, Simpson) and beta diversity (PCA, PERMANOVA) were calculated using the vegan^66^ and prcomp packages in R. Microbial gene expression was collapsed to the genus level by aggregating TPM values for the top 10 genera across all samples. VIRGO2 annotations included bacteriophage and viral transcripts, allowing for quantification of sexually transmitted infections (STIs), viruses, and phages over time. Expression differences between groups and timepoints were assessed using Wilcoxon tests.

### Differential gene expression and pathway enrichment

Microbial and host RNA-seq data were filtered to retain genes present in ≥ 90% of samples within at least one response group (recurrent vs. nonrecurrent BV). Data were normalized using the trimmed mean of M-values (TMM) and log2 counts-per-million (CPM). Differential expression analysis was conducted using the limma-voom pipeline^67,68^. Microbial DEGs were subjected to KEGG^69^ pathway enrichment using enricher and term2gene, with all expressed genes as background. Host pathway enrichment was performed using gprofiler2::gost against the GRCh38 human reference.

### Cytokine quantification and correlation analysis

Twelve cytokines were measured in cervicovaginal lavage fluid using a custom Luminex assay (DiaSorin) (GM-CSF, IFN-γ, IL-1β, IL-2, IL-5, IL-6, IL-8, IL-10, IL-17A, TNF-α, MIP-1α, MIP-1β). Between- and within-group comparisons were made using Wilcoxon tests. Spearman correlations were computed between cytokine concentrations and microbial DEGs, host DEGs, and STI markers at each timepoint. Significant correlations (FDR-adjusted p < 0.05) were visualized in heatmaps, stratified by taxonomic origin and COG functional category.

### Bayesian modeling

Bayesian hierarchical logistic regression models were constructed using the brms^70^ package to evaluate the influence of microbial, host, and cytokine variables on BV recurrence. Predictors included the most significant microbial genera, cytokine levels, and host genes. All predictors were z-transformed to allow comparison of effect sizes. Models included participant-level random intercepts and employed weakly informative or noninformative priors. MCMC^71^ sampling was performed using 12 chains (3,000 iterations each; 500 warm-up), with adapt_delta = 0.95 and max_treedepth = 25. Model convergence was confirmed with R-hat ≤ 1.01.

Bayesian mediation analyses were implemented to assess direct and indirect effects of exposures (e.g., *Lactobacillus*, *Gardnerella*, intravaginal product use) via mediators (e.g., CEACAM5, IL6) on the outcome of BV recurrence. Simple mediation models were fit using joint likelihoods with set_rescor = FALSE. Parallel and sequential mediation models did not converge due to sample size constraints and were not further interpreted. Indirect, direct, and total effects were estimated from posterior samples, and changes in explained variance (ΔR^2^) were computed by comparing Bayesian R^2^ between full and reduced models.

## Supplementary Material

Supplementary Files

This is a list of supplementary files associated with this preprint. Click to download.

- SupplementalData.docx

## Data Availability

All analyses were conducted in R (v4.3.1). Data wrangling and visualization were performed using tidyverse^72^, dplyr^73^, ggplot2^74^, ggdist^75^, patchwork^76^, and gt^77^. Custom code and full analysis workflows are available via the Klatt Lab GitHub repository.
